# Analysis of medical impoverishment and its influencing factors among China's rural near-poor, 2016–2020

**DOI:** 10.3389/fpubh.2024.1412536

**Published:** 2024-05-16

**Authors:** Qiwei Feng, Yi Che, Shuying Yi, Ying Wang, Wen Chen, Xinbin Xia

**Affiliations:** ^1^School of Humanities and Management, Hunan University of Chinese Medicine, Changsha, Hunan, China; ^2^Key Laboratory of Vascular Biology and Translational Medicine, Medical School, Hunan University of Chinese Medicine, Changsha, China

**Keywords:** medical impoverishment, near-poor, impoverishing health expenditures, relative poverty, conditional fixed-effects multinomial logit model, influencing factors, rural China

## Abstract

**Objective:**

This study investigates the determinants of medical impoverishment among China's rural near-poor, aiming to enhance public health services and establish preventative and monitoring systems.

**Methods:**

Using China Family Panel Studies and World Bank methods, we categorized rural populations and calculated their 2020 Poverty Incidence (PI) and Poverty Gap (PG), with impoverishing health expenditures (IHE) as the primary indicator. We analyzed the data from 2016 to 2020 using a conditional fixed-effects multinomial logit model and 2020 logistic regression to identify factors influencing medical impoverishment risk.

**Results:**

(1) In 2020, the near-poor in China faced a PI of 16.65% post-health expenditures, 8.63 times greater than the non-poor's PI of 1.93%. The near-poor's Average Poverty Gap (APG) was CNY 1,920.67, notably surpassing the non-poor's figure of CNY 485.58. Health expenses disproportionately affected low-income groups, with the near-poor more prone to medical impoverishment. (2) Disparities in medical impoverishment between different economic household statuses were significant (*P* < 0.001), with the near-poor being particularly vulnerable. (3) For rural near-poor households in China, those with over six members faced a lower risk of medical impoverishment compared to those with three or fewer. Unmarried individuals had a 7.1% reduced risk of medical impoverishment relative to married/cohabiting counterparts. Unemployment was associated with a 9% increased risk. A better self-rated health status was linked to a lower probability of IHE, with the “very healthy” reporting a 25.8% lower risk than those “unhealthy.” Chronic disease sufferers in the near-poor and non-poor categories were at an increased risk of 12 and 1.4%, respectively. Other surveyed factors, including migrant status, age, insurance type, gender, educational level, and recent smoking or drinking, were not statistically significant (*P* > 0.05).

**Conclusion:**

Rural near-poor in China are much more susceptible to medical impoverishment, influenced by specific socio-economic factors. The findings advocate for policy enhancements and health system reforms to mitigate health poverty. Further research should extend to urban areas for comprehensive health poverty strategy development.

## 1 Introduction

In “Transforming Our World: The 2030 Agenda for Sustainable Development,” the United Nations outlined 17 Sustainable Development Goals (SDGs), the foremost of which targets the elimination of poverty in all its forms globally by 2030 (1). Nonetheless, the advent of the COVID-19 pandemic has significantly impeded the consistent progress made in poverty reduction over the past 30 years. Projections suggest that, by the year 2030, an estimated 575 million individuals will still subsist in conditions of extreme poverty, and merely a third of nations are on track to halve their poverty rates (2). Eradicating poverty continues to be one of the most pressing global challenges.

In contrast to other poverty-inducing factors, illness tends to result in sustained impoverishment (3). Research indicates that 51.63% of rural impoverished populations in China link their poverty to familial health issues, identifying medical impoverishment as a fundamental cause of rural poverty in the country (4). The exorbitant health expenditures stemming from illness have the potential to rapidly exhaust family savings, leading to severe economic distress. Additionally, illness diminishes a patient's productivity and learning capacity, undermining their competitiveness in the labor market and future earning prospects (5). Consequently, families in poverty are susceptible to an insidious “illness-poverty” cycle, wherein illness precipitates financial loss and further constrains access to healthcare, exacerbating their economic and health conditions (6). It is thus crucial to pinpoint the pivotal factors contributing to medical impoverishment to disrupt this deleterious cycle.

Reports from the World Health Organization (WHO) and the World Bank indicate that from 2000 to 2019, there was an 80% decline in the global proportion of individuals who faced impoverishing out-of-pocket health expenditures while under the extreme poverty threshold. Conversely, the proportion of individuals experiencing impoverishing health expenditures (IHE) under the relative poverty threshold saw a 42% increase during the same timeframe (7). These findings suggest a shift in focus toward those near or slightly above the poverty line (PL) within the sector of health poverty alleviation. The near-poor population is defined as households with annual per capita incomes that fall between one and two times the local poverty threshold (8). Typically, these individuals do not have a stable income and are subjected to greater health, economic, and social pressures compared to the general populace (9). Unfortunately, they often fail to gain from social support and policy measures. An oversight in health poverty alleviation policies and the deficiencies of the healthcare security system intensify the vulnerability of this demographic to poverty due to health-related expenses, positioning them as a central group susceptible to medical impoverishment.

In recent years, scholars have primarily examined the impact of household characteristics, individual behavior, and structural factors on the risk of falling into medical impoverishment. Factors such as household size (10), whether one belongs to a migrant group (11, 12), the presence of children (13, 14) or older adult individuals (15), employment status (16), the nature of employment, household asset structure, family composition (17), and chronic illness or disability (4, 18) can all affect the risk of health poverty among respondents. Unhealthy behaviors like poor diet, smoking, or a lack of physical activity (3) can lead to lower levels of health, which are significantly associated with poverty. Structural factors such as the local economic development and social security systems (19–23), the risk of fuel scarcity and climate (24), social transfers (25), and other related constructs like social cohesion, social capital, and social exclusion (11) also impact health poverty. Furthermore, considerable literature has begun to explore relative standards of health poverty, such as using specific proportions of the median income to calculate relative poverty thresholds (26, 27). It is argued that relative poverty standards should be multidimensional, incorporating a variety of criteria including health, education, family structure, economic status, and labor market levels (28, 29). This body of work emphasizes the importance of preventing medical impoverishment in poverty alleviation efforts and explores various factors influencing medical impoverishment, providing a solid foundation for this research. However, there are limitations: firstly, most studies focus on specific populations such as children, the older adult, migrant populations (12), and particular ethnic groups, with less attention to those on the cusp of poverty or the near-poor; secondly, poverty incidence (PI) and poverty gap (PG) are often measured based on catastrophic health expenditures (CHE), using the proportion of out-of-pocket health spending to total household income; thirdly, investigations into the factors influencing medical impoverishment are frequently based on cross-sectional data, with fewer studies constructing panel data models for longitudinal comparison and robustness checks.

Based on the preceding discussion, this study posits that governance of medical impoverishment should begin with the perspective of relative poverty, focusing particularly on the near-poor population in impoverished areas. Analyzing the factors contributing to medical impoverishment among these individuals can aid in the development of precise and effective health poverty alleviation measures targeted at high-risk groups, thus enhancing the efficiency and effectiveness of poverty alleviation initiatives (30). The specific approaches are as follows: (1) The study targets the near-poor population in rural China. Given that the economic development level in rural areas is generally lower than that in urban areas, rural regions have always been the focal point of China's poverty eradication efforts. Although China eradicated absolute poverty by the end of 2020, medical impoverishment and the risk of falling back into poverty remain key challenges in poverty alleviation. (2) From the perspective of relative poverty, the study utilizes the income median ratio method to define the poverty and near-poor lines, categorizing the population into poor, near-poor, and non-poor groups. It employs the World Bank's recommended method for measuring IHE to assess the impoverishing impact of health expenditures and to understand the current status of IHE among the rural near-poor population. (3) The study uses logit models and conditional fixed-effects multinomial logit models to focus on the relationship between household economic status and medical impoverishment. It analyzes the factors influencing the occurrence of medical impoverishment among the national rural near-poor population. China's achievements in health poverty alleviation can serve as a model for other relatively underdeveloped countries and regions. This research provides targeted evidence support and poverty alleviation recommendations for the prevention of large-scale poverty relapse, the establishment of long-term mechanisms to address relative poverty, and the construction of enduring early warning, monitoring, and safeguard measures.

## 2 Materials and methods

### 2.1 Data sources and preprocessing

This study utilizes data from the China Family Panel Studies (CFPS), a national, longitudinal social survey conducted by the Institute of Social Science Survey at Peking University. The CFPS is administered biennially, encompassing 25 provinces, autonomous regions, and municipalities, representing over 95% of the Chinese population. It captures multifaceted information on Chinese households and their members, including social, economic, demographic, educational, and health aspects. The survey employs a multi-stage, multi-level probability proportional sampling design and provides various sample weights, such as cross-sectional weights for individuals or households and longitudinal tracking weights.

To ensure the representativeness of the samples, all analyses in this study were weighted according to the corresponding weights based on the analytical objectives. The CFPS provided standardized weights for the years 2018 and 2020, with the 2020 weights further refined into non-response weights and post-stratification weights. However, all weights for the year 2016 were unstandardized. For comparative analysis purposes, we standardized the weights concerning the year 2016 in our research, while for the 2020 data, we employed post-stratification weights to enhance accuracy.

To further ensure the representativeness of the sample, given the presence of “not applicable” and “missing” data in the raw CFPS dataset, we prioritized the imputation of missing values and outliers using data from different sub-databases provided by the CFPS for the same year. For data that could not be found, if the corresponding values in adjacent years did not change significantly, we imputed the values using data from the adjacent years. We identified the samples using the household identification number and individual identifier provided by the CFPS. We matched the family economic database and the individual database (for those aged 16 and above) from the years 2016, 2018, and 2020 both cross-sectionally and longitudinally. After imputation and weighting, we excluded missing and outlier values that could not be imputed, thereby obtaining all the variable information and data required for the study.

Specifically, we obtained three cross-sectional databases from the family economic databases for the years 2016, 2018, and 2020, which were used to calculate the PLs for the corresponding years and to classify the population accordingly. The weights applied in this step were the cross-sectional household weights for each respective year. The number of observations in the 2016 database was 13,998, with a weighted sample size of 13,995; both 2018 and 2020 had consistent sample sizes before and after weighting, with 14,218 and 11,620 samples, respectively.

After calculating the PL using the family economic database, we constructed two databases to delve into the factors influencing medical impoverishment among the near-poor population. The first is a cross-sectional database for the year 2020, with 9,796 observations. After weighting with post-stratification standardized individual cross-sectional weights, the total sample size amounted to 8,072. Utilizing this database, we estimated the PI and PG for 2020 as a case study and developed logistic regression models to analyze the factors influencing medical impoverishment among the near-poor. The second database is a three-wave unbalanced panel dataset containing 32,433 observations (13,457 for 2016, 10,917 for 2018, and 8,059 for 2020). This panel data, analyzed using post-stratification standardized individual panel weights, was employed to conduct descriptive analyses and to construct conditional fixed-effects multinomial logit models, exploring longitudinally the critical factors affecting medical impoverishment among the near-poor. After weighting, the weighted sample sizes for each wave were 10,324 for 2016, 8,373 for 2018, and 6,160 for 2020. All data processing and analysis were conducted using Stata 17.0.

### 2.2 Relative poverty line and population stratification

In this study, we used the variable fincome1_per from the CFPS family economic database to represent annual per capita family income and employed the post-stratification weight variable from the same database to weight population numbers and incomes. From the perspective of relative poverty, the poverty line (PL) was set according to the median income proportion method. Taking into account international experience and regional economic disparities during China's 14th Five-Year Plan period, we determined 40% of the median annual per capita family income in the sample regions as the PL (10). Following the standards of the U.S. Census Bureau, individuals with annual per capita family incomes below the local PL were classified as poor, those with incomes between one and two times the PL were classified as near-poor, and those with incomes more than twice the PL were classified as non-poor. For ease of subsequent analysis, we created a new variable, “family economic status,” to distinguish between poor, near-poor, and non-poor samples.

### 2.3 Measurement of impoverishing health expenditures, poverty incidence, and poverty gap

This study employs the IHE measure recommended by the World Bank to estimate the impoverishing effect of health expenditures (31). The impoverishing effect is assessed by comparing changes in the poverty status of individuals before and after incurring health expenditures. The extent and depth of poverty, ensuing from health expenditures, are respectively represented by the poverty incidence and poverty gap.

The poverty incidence (PI) represents the proportion of individuals living in poverty relative to the total population. It reflects the prevalence or occurrence of poverty within a demographic by indicating the share of the population that is impoverished. The PL signifies the local relative poverty threshold. If individual *i*'s income is below PL, *P*_*i*_ equals 1, signifying poverty; otherwise, *P*_*i*_ equals 0, indicating no poverty. Based on prior literature (31, 32), the formula for calculating poverty incidence is as follows (where *N* denotes the total population size):


(1) $$PI=\frac{1}{N}\sum_{i=1}^{N}P_{i}$$

The concept of the poverty gap (PG), alternatively termed as the poverty shortfall, quantifies the extent to which the income of impoverished individuals falls beneath the relative PL. This measure provides an economic differential view on the severity of poverty and delineates the disparity between an individual or group's present condition and the target of poverty eradication. The *income*_*i*_ denotes the income level of resident *i*. The PG is further categorized into the average poverty gap (APG) and the relative poverty gap (RPG). The APG is the sum of the individual poverty gaps within a population, divided by the total population, offering a reflection of the aggregate poverty level. Conversely, the RPG is calculated by dividing the sum of the population's PGs by the number of impoverished individuals, thereby illustrating the extent of poverty within this group. In accordance with prior literature (31, 32), these can be mathematically represented as follows (where N denotes the population size):


(2) $$\begin{array}{l} PG_{i}=P_{i}\times \left(PL-income_{i}\right) \end{array}$$

(3) $$\begin{array}{l} APG=\frac{1}{N}\overset{N}{\underset{i=1}{\sum }}PG_{i} \end{array}$$

(4) $$\begin{array}{l} RPG=\overset{N}{\underset{i=1}{\sum }}PG_{i}/\overset{N}{\underset{i=1}{\sum }}P_{i} \end{array}$$

### 2.4 Analysis of conditional fixed-effects multinomial logit models on medical impoverishment

To address potential omitted variable bias caused by unobservable individual heterogeneity and to further investigate the factors influencing the risk of falling into medical impoverishment among the near-poor, this study constructed a three-wave unbalanced panel dataset using individual-level data from the CFPS for the years 2016, 2018, and 2020. When analyzing multi-level sampling panel data using Stata 17.0 software, the svy command must be employed. Additionally, the dependent variable is a binary variable indicating “whether an individual experiences medical impoverishment,” and two-way fixed effects of time and individual often exist in the field of health economics. Currently, the conditional fixed-effects multinomial logit model is the only model in Stata software that can simultaneously satisfy these conditions. This model is suitable for panel data with a multinomial dependent variable and can handle fixed effects. Although the dependent variable in this study is dichotomous, it is technically feasible to treat a binary variable as a special case of a multinomial variable, as supported by the literature (33, 34). To further ensure the accuracy of the model and results, we constructed different models, such as random effects and individual fixed effects, by adding or removing variables to test the robustness of the results.

The baseline regression model is as follows:


(5) $$\begin{array}{c} \log\left(\frac{P\left(Y_{it}=1\right)}{P\left(Y_{it}=0\right)}\right)=\delta_{1}FES_{it}+\delta_{2}x_{1it}+\delta_{3}x_{2it}+\ldots \\ +\delta_{K}x_{\left(K-1\right)it}+\mu_{i}+\mu_{t}+\varepsilon_{it} \end{array}$$

The dependent variable *Y*_*it*_ denotes whether an individual has fallen into medical impoverishment. This condition is met when a resident's income, after health expenditures, dips below the relative PL, signifying IHE leading to impoverishment (35). *P*(*Y*_*it*_ = 1) denotes the probability that individual *i* falls into medical impoverishment at time *t*, while *P*(*Y*_*it*_ = 0) indicates the probability that individual *i* does not experience medical impoverishment at time *t*. The primary explanatory variable *FES*_*it*_ refers to the economic status of individual *i*'s household in year *t*. The variables *x*_1*it*_, *x*_2*it*_, …, *x*_(*K*−1)*it*_ represent various control variables, including age, gender, marital status, education level (36), employment status (37), household size (10), type of medical insurance coverage (19), migrant status (12), smoking and drinking habits (3), self-rated health status (38), and presence of chronic diseases (39, 40), with *K* representing the total number of control variables plus one. The coefficient δ_*K*_ reflects the impact of the corresponding variable *x*_(*K*−1)*it*_ on the likelihood of becoming medical impoverishment, where δ_1_ specifically represents the impact of family economic status. μ_*i*_ denotes individual effects, μ_*t*_ represents time effects, and ε_*it*_ is the error term.

### 2.5 Analysis of the factors influencing impoverishing health expenditure

As the dependent variable “whether an individual experiences medical impoverishment” is a binary variable, we constructed a binary logistic regression model (41) based on the CFPS 2020 data. We selected the near-poor and non-poor populations in 2020 as samples and compared them to thoroughly explore the factors leading to medical impoverishment among the near-poor population. To ensure the validity of the model, we conducted collinearity tests and chi-square tests before constructing the model. Only independent variables that passed these tests were included in the regression model. Since the logit regression coefficients represent the change in the log odds of the dependent variable for each unit change in the independent variable, which is not conducive to subsequent discussion and interpretation, we further analyzed the marginal effects (dy/dx). The marginal effects indicate the change in the probability of the dependent variable occurring for each unit change in the independent variable, making it more convenient to interpret the model results. The logistic regression model is presented as follows:


(6) $$\begin{array}{l} \log\left(\frac{P\left(Y_{it}=1\right)}{P\left(Y_{it}=0\right)}\right)=\beta_{0}+\sum \beta_{i}x_{i} \end{array}$$

The dependent variable *Y*_*it*_ is consistent with Equation (5). The independent variable *x*_*i*_ includes a range of factors: age, gender, marital status, level of education, employment status, household size, migrant status, type of medical insurance coverage, smoking and drinking habits, self-rated health status, and chronic disease conditions. The coefficient β_*i*_ corresponds to each *x*_*i*_, with β_0_ representing the constant term.

Details on the variable assignments are provided in Supplementary Table 1.

## 3 Results

### 3.1 Descriptive statistics

Supplementary Table 1 presents the weighted baseline characteristics of the rural Chinese population for the years 2016, 2018, and 2020, after adjusting for individual panel weights. In terms of sociodemographic characteristics: despite using a relative PL to differentiate between the poor and the near-poor, the proportion of the rural poor population in China steadily declined across the periods, accounting for 40.84, 35.05, and 32.38% in the respective years, a total decrease of 8.45%. However, the percentage of the near-poor population remained around 20%, with 19.42% in 2016, 21.06% in 2018, and 20.5% in 2020. The population of older adult individuals aged 65 and above incrementally increased, from 17.7% in 2016 to 21.09% in 2020. Changes in gender ratio were not significant, yet the absolute difference in proportions widened, rising from 0.67% in 2016 to 2.39% in 2020. Marital status showed no substantial change, but there was a noticeable increase in single individuals in 2020 (a 2.06% increase from 2016), coupled with a decline in the total number of married and cohabiting individuals. The proportion of the population with elementary education or less significantly decreased by 10.52% points from 2016 to 2020, still accounting for 47.71%. Meanwhile, those with secondary education rose steadily, and the group with higher education expanded by nearly 5% points over 4 years. The employed population slightly increased, with just a 0.6% rise from 2016 to 2020. The proportion of employed individuals remained consistently lower than that of the population under 65 years of age, but the gap narrowed, with the employed population being 7.92, 4.42, and 3.93% less than the under-65 population in the years 2016 to 2020, respectively. The number of migrants showed a significant decline, from 22.11% in 2016 to 10.77% in 2020. Post-2018, there was a rise in households with three or fewer members, a decrease in four to five-member households, and no significant changes in households with six or more members.

Examining individual health behaviors over the three periods, the proportion of individuals who smoked in the past month, consumed alcohol more than three times per week, or the presence of chronic diseases did not follow a clear trend, fluctuating around 29, 17, and 16%, respectively, with each peaking in 2018 before declining in 2020. The percentage of individuals who perceived themselves as relatively healthy (including relatively healthy, very healthy, and extremely healthy) steadily increased, rising by 8.5% from 2016 to 2020.

Regarding structural factors, the proportion of individuals without medical insurance incrementally increased, with a modest growth of 3% points over the periods. The number of people purchasing basic medical insurance for urban and rural residents decreased annually, falling by 6.62% in 2020 compared to 2016, whereas the number of individuals opting for other types of medical insurance saw an annual increase, rising by 3.92% by 2020 compared to 2016.

### 3.2 The impoverishing impact of residents' health expenditures

Using the household economic database and the standardized cross-sectional weights for each year, we established the PL for each year (see Table 1), which allowed us to conduct an in-depth analysis of the impoverishing effect of health expenditures using the 2020 cross-sectional individual data.

**Table 1:** Poverty lines and near-poverty lines from 2016 to 2020.

**Variables**	**2016**	**2018**	**2020**
Median per capita household income	15,000	20,000	22,098
Poverty line (40% of median income)	6,000	8,000	8,839
Near-poverty line (twice the poverty line)	12,000	16,000	17,678

All monetary values are in Chinese Yuan (CNY).

Table 2 presents the impoverishing impact of health expenditures in 2020 across different economic groups. After accounting for health expenditures, the PI of the near-poor was 16.65%, which is ~8.63 times higher than that of the non-poor population (1.93%). Considering the PG, the APG for the poor before and after health expenditures was 12,486.34 Chinese Yuan (CNY) and CNY 14,487.63, respectively, with health expenditures deepening their poverty by CNY 2,001.29. The APG post-health expenditures for the near-poor was CNY 1,920.67, substantially higher compared to the non-poor at CNY 485.58. In terms of the RPG, the figures for the near-poor and the poor were CNY 11,537.35 and CNY 14,487.63, respectively, showing little difference; however, the impoverishing effect of health expenditures on individuals at risk of falling back into poverty increased progressively among the poor, near-poor, and non-poor groups, amounting to CNY 2,001.29, CNY 11,537.35, and CNY 25,132.52, respectively.

**Table 2:** The impoverishing impact of health expenditures on populations with different economic statuses.

**Indicator**	**Poverty incidence rate (%)**			**APG**			**RPG**		
	**Poor**	**Near-poor**	**Non-poor**	**Poor**	**Near-poor**	**Non-poor**	**Poor**	**Near-poor**	**Non-poor**
Health expenditure before	100	0	0	12,486.34	0	0	12,486.34	0	0
Health expenditure after	100	16.65	1.93	14,487.63	1,920.67	485.58	14,487.63	11,537.35	25,133
Impoverishing impact	0	16.65	1.93	2,001.29	1,920.67	485.58	2,001.29	11,537.35	25,133

All monetary values are in Chinese Yuan (CNY). The Average Poverty Gap (APG) is the sum of the individual poverty gaps within a population, divided by the total population, offering a reflection of the aggregate poverty level. Conversely, the Relative Poverty Gap (RPG) is calculated by dividing the sum of the population's poverty gaps by the number of impoverished individuals, thereby illustrating the extent of poverty within this group.

**Figure 1:**
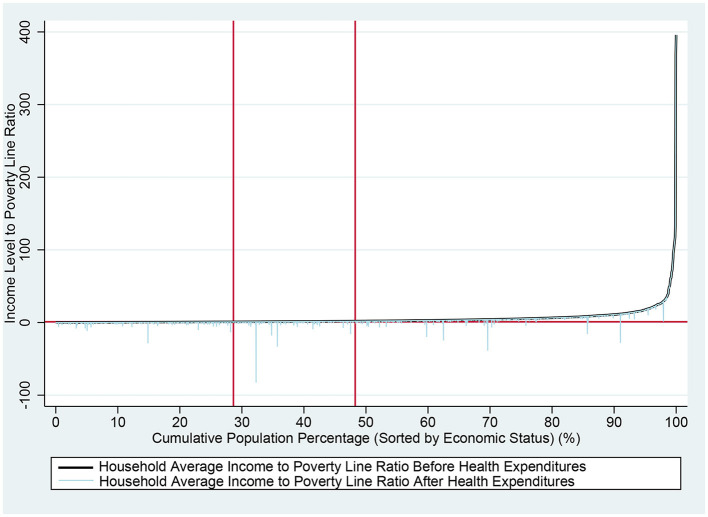
Comparison of rural residents' income before and after health expenditures nationwide. The horizontal axis represents the cumulative percentage of the population sorted by income level, while the vertical axis shows the ratio of individual income to the PL, which is expressed as a multiple of the PL. A horizontal line in the charts indicates the income level equivalent to the PL. The two vertical marker lines, from left to right, indicate the PL and the near-poverty line, respectively. The rising curves from left to right depict individual incomes before and after health expenditures; the smoother upper curve represents pre-expenditure income, while the stepped curve below reflects post-expenditure income, with the drops indicating the amount spent on health. A drop below the line representing the PL indicates IHE.

**Figure 2:**
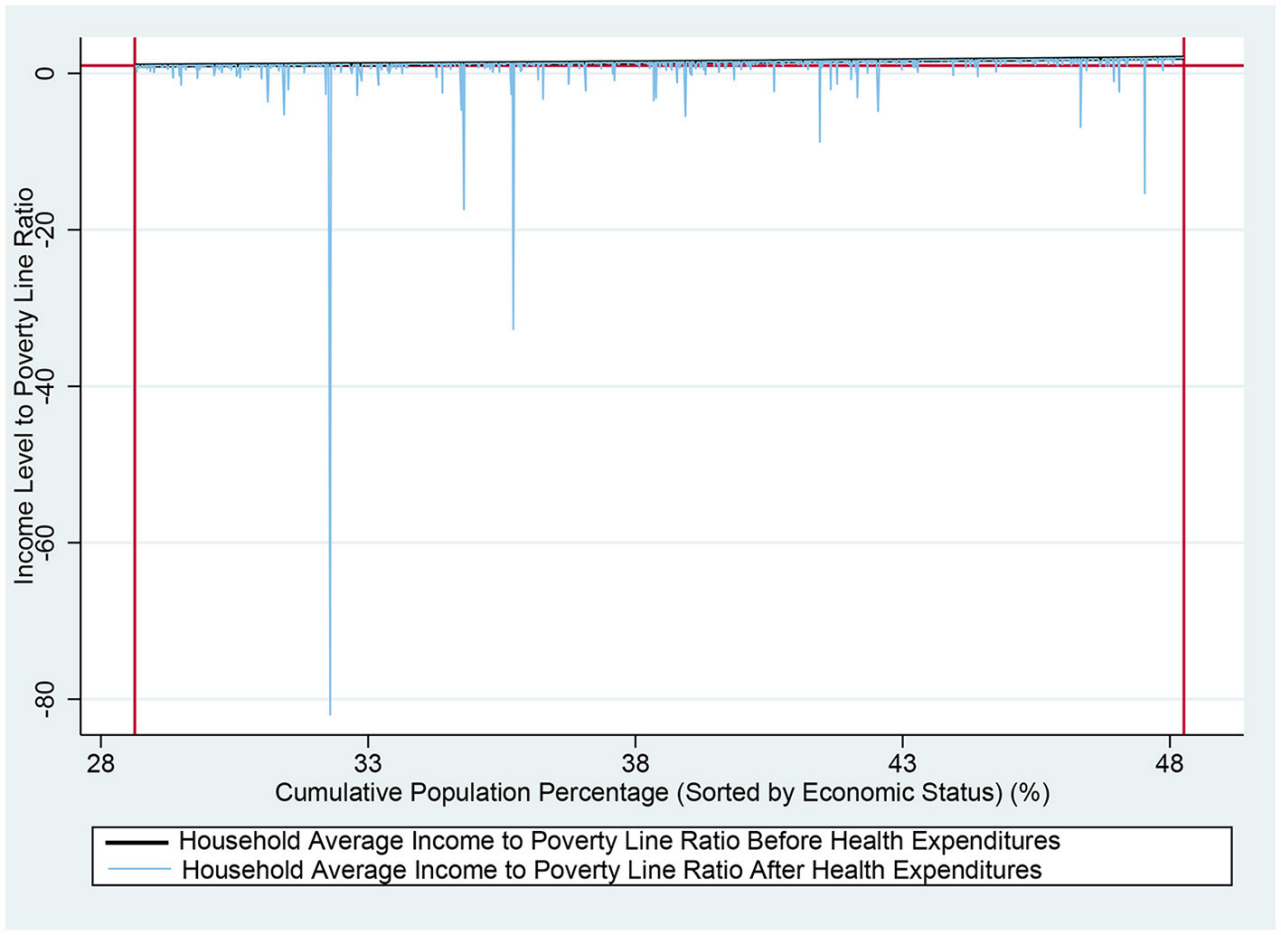
Comparison of income among near-poor rural residents before and after health expenditures nationwide. The horizontal axis represents the cumulative percentage of the population sorted by income level, while the vertical axis shows the ratio of individual income to the PL, which is expressed as a multiple of the PL. A horizontal line in the charts indicates the income level equivalent to the PL. The two vertical marker lines, from left to right, indicate the PL and the near-poverty line, respectively. The rising curves from left to right depict individual incomes before and after health expenditures; the smoother upper curve represents pre-expenditure income, while the stepped curve below reflects post-expenditure income, with the drops indicating the amount spent on health. A drop below the line representing the PL indicates IHE.

**Figure 3:**
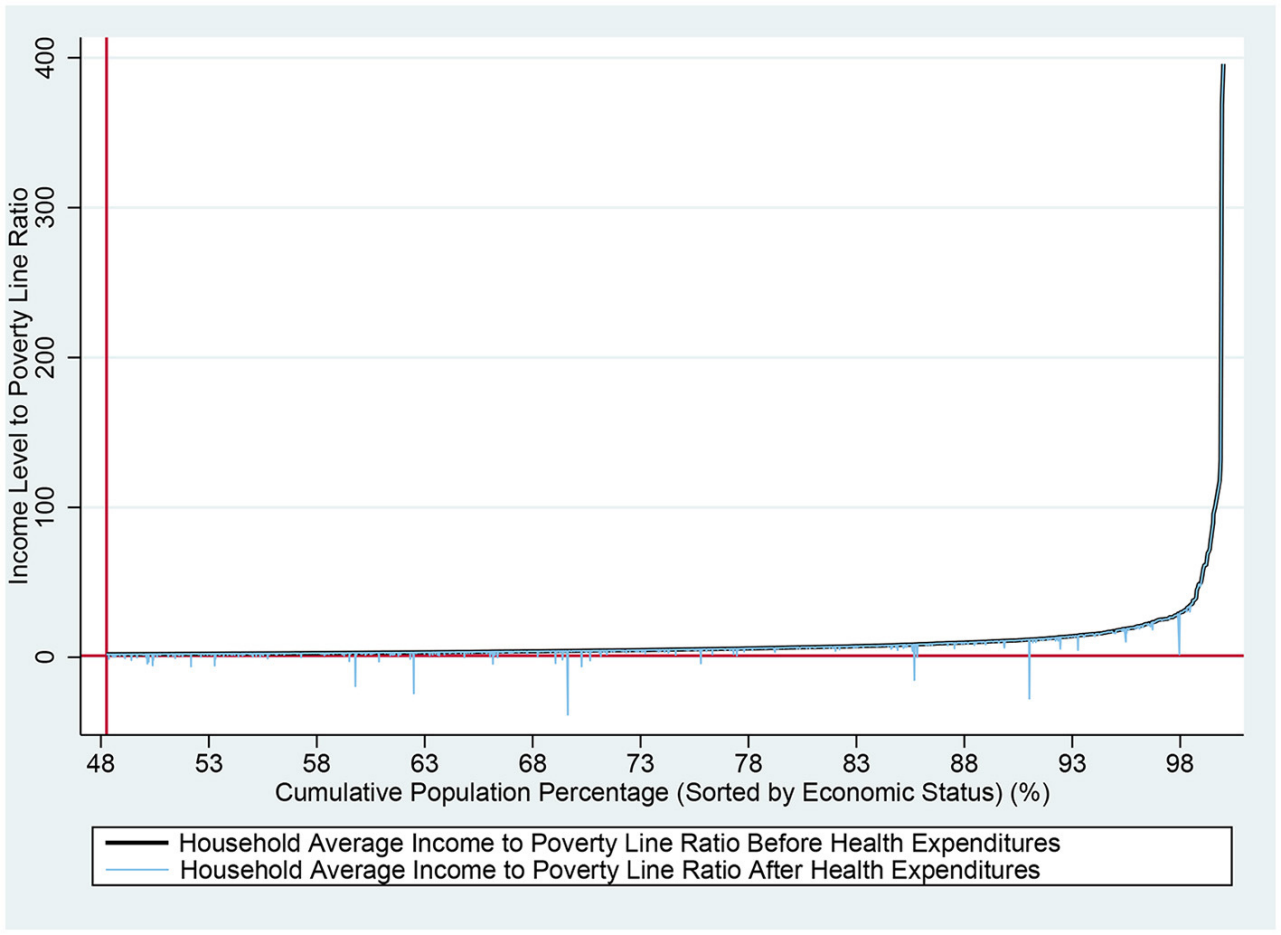
Comparison of income among non-poor rural residents before and after health expenditures nationwide. The horizontal axis represents the cumulative percentage of the population sorted by income level, while the vertical axis shows the ratio of individual income to the PL, which is expressed as a multiple of the PL. A horizontal line in the charts indicates the income level equivalent to the PL. The vertical marker line indicates the near-poverty line. The rising curves from left to right depict individual incomes before and after health expenditures; the smoother upper curve represents pre-expenditure income, while the stepped curve below reflects post-expenditure income, with the drops indicating the amount spent on health. A drop below the line representing the PL indicates IHE.

Comparative income charts (Figures 1–3) before and after health expenditures provide a visual analysis of the impoverishing impact these expenditures have on families with different economic statuses. The horizontal axis represents the cumulative percentage of the population sorted by income level, while the vertical axis shows the ratio of individual income to the PL, which is expressed as a multiple of the PL. A horizontal line in the charts indicates the income level equivalent to the PL, and two vertical lines respectively mark the poverty and near-poverty lines. The rising curves from left to right depict individual incomes before and after health expenditures; the smoother upper curve represents pre-expenditure income, while the stepped curve below reflects post-expenditure income, with the drops indicating the amount spent on health. A drop below the line representing the PL indicates IHE. These charts clearly illustrate the frequency and magnitude of health expenditures and the distribution of economic status among different families, thus facilitating a better analysis of the impoverishing effects of health expenditures. Figures 1–3 reveal that the impoverishing impact of health expenditures is predominantly concentrated among low-income groups, with the near-poor shown to be more susceptible to medical impoverishment than their non-poor counterparts.

### 3.3 Conditional fixed-effects multinomial logit models analysis of medical impoverishment

Supplementary Table 2 presents the regression results from the conditional fixed-effects multinomial logit models. Given the categorical nature of the dependent variable and the complexity of the panel data, which required weighting, and the fact that weighted models are incompatible with various tests such as the Hausman test, we conservatively chose the conditional fixed-effects multinomial logit model with two-way fixed effects as our baseline model (Model 1 in Supplementary Table 2), in line with common practice in health economics and policy. To assess the model's robustness, we expanded it by adding variables. Prior to formal modeling, chi-square tests were conducted for all variables, which all passed and were thus included in the baseline model construction. The baseline model showed statistical significance, with an *F*-value of 3,723.55 and *P* < 0.001. Model 2 incorporates individual fixed effects, Model 3 includes only the core explanatory variable “household economic status,” Model 4 extends Model 3 by controlling for time fixed effects, and Models 5 and 6 sequentially introduce demographic and behavioral characteristic variables on top of Model 4. The core explanatory variable “household economic status,” using the near-poor as a reference, had a significantly negative coefficient for the non-poor (*P* < 0.001), indicating that the non-poor are less likely to fall into medical impoverishment compared to the near-poor. This primary finding remained consistent across different model specifications. Additionally, according to the baseline model, household size, age, marital status, self-rated health, and the presence of chronic disease all had a significant impact on the likelihood of falling into medical impoverishment (*P* < 0.05).

### 3.4 Logit regression analysis of factors influencing medical impoverishment

To further explore the factors that lead to medical impoverishment among the near-poor population, we constructed logistic regression models using cross-sectional data from 2020. Model 1 targeted the near-poor population, while Model 2 focused on the non-poor population. Supplementary Table 3 presents the results of the logit regression analysis for medical impoverishment among rural residents in China in 2020. Variables such as “type of medical insurance coverage” and “alcohol consumption 3 Times a week in the past month” were not included in the model construction, as they did not pass the univariate analysis for either sample group. For the sake of comparison, some other independent variables were included in the models even if they did not pass the chi-square test for one of the sample groups. All models were statistically significant, with *P* < 0.001, indicating that the models were meaningful.

Supplementary Table 3 reveals that for the rural near-poor population, households with more than six members are less likely to experience medical impoverishment compared to smaller families of three members or fewer, though the marginal effect of household size is not pronounced. Additionally, household size does not significantly impact the rural non-poor population. Compared to the older adult aged 65 and above, the probability of falling into medical impoverishment among the non-poor under the age of 35 decreases by 2.6%, but age does not significantly affect the near-poor. For the near-poor, single individuals are 7.1% less likely to become medical impoverishment compared to those who are married or cohabitating, and individuals not currently employed are 9% more likely compared to those who are employed; however, employment and marital status have no significant impact on the non-poor population. Self-rated health status significantly impacts the risk of medical impoverishment among rural residents. Those who consider themselves in good health are less likely to incur IHE, with the near-poor who view themselves as very healthy having a 25.8% lower probability of falling into medical impoverishment compared to those who perceive themselves as unhealthy. This probability also decreases for the non-poor, though by no more than 5%. Relative to those without chronic diseases, the likelihood of the near-poor and non-poor with chronic diseases falling into medical impoverishment increases by 12 and 1.4%, respectively. The probability for non-poor migrant populations to fall into medical impoverishment increases by 1.7% compared to non-migrant non-poor populations, but migrant status has no significant effect on the near-poor. Lastly, gender, education level, and smoking in the past month have no impact on either group.

## 4 Discussion

The rural near-poor population in China faces a high risk of falling into medical impoverishment. Descriptive analysis indicates that even when poverty is measured using relative standards, China has achieved commendable results in its poverty alleviation efforts. However, the near-poor population remains substantial, accounting for about 20% of the rural total population. The analysis of the impoverishing effect of health expenditures reveals that the impact is predominantly concentrated among the low-income groups. The probability of the near-poor falling into medical impoverishment is 8.63 times greater than that of the non-poor, with the APG amounting to CNY 1920.67 and CNY 485.58, respectively, indicating a higher likelihood of medical impoverishment among the near-poor compared to the non-poor. The conditional fixed-effects multinomial logit model analysis also demonstrates that the core variable “household economic status” significantly affects the likelihood of falling into medical impoverishment, with the near-poor more likely to experience this compared to the non-poor population (*p* < 0.001). These findings are consistent with previous research (9, 42, 43), suggesting a significant disparity between the near-poor and non-poor populations. Policymakers should focus on the near-poor population to reduce their risk of poverty.

Age is not a key factor affecting the risk of medical impoverishment among the rural near-poor population. Results from both the conditional fixed-effects multinomial logit model and the cross-sectional logit model analysis for the year 2020 show that age has a significant impact on the likelihood of falling into medical impoverishment, consistent with prior research (4). However, a more detailed analysis of the factors leading to medical impoverishment among different groups reveals that age primarily affects the non-poor rather than the near-poor, which differs from some previous studies (44). Age has different effects on the near-poor and non-poor populations, which may be due to significant differences in their socioeconomic support systems. The non-poor population typically has a more comprehensive economic support system, making the impact of age on health and economic burden more pronounced. In contrast, the near-poor population is on the margins of socioeconomic status, and the influence of age may be overshadowed by other socioeconomic factors. Furthermore, the near-poor population faces issues such as a lack of access to medical resources and social network support, which may further weaken the impact of age as a health risk factor. Although our classification of individuals into near-poor, poor, and non-poor groups is based on per capita measurements, and individuals may offset the risk of falling into medical impoverishment with financial help from relatives, our results remain credible. This credibility is due to our income measurement already accounting for financial assistance from relatives, friends, and socio-governmental support.

Gender, education level, type of medical insurance coverage, and behaviors such as smoking and drinking do not significantly affect the risk of medical impoverishment among the near-poor population. According to the cross-sectional logit regression results, gender does not have a significant impact on whether individuals in the near-poor category fall into medical impoverishment. This suggests that both males and females may face similar health risks and economic vulnerabilities. The coefficient for the education level variable is negative for both near-poor and non-poor groups, suggesting that higher education levels are associated with a reduced likelihood of medical impoverishment, but this effect is not statistically significant. This finding may be attributed to the overall low education level and minimal internal differences within this group. Analyses of both panel and cross-sectional data indicate that drinking more than three times a week or smoking during the past month does not significantly influence the risk of falling into medical impoverishment. This finding is somewhat inconsistent with previous research, which has shown that smoking can exacerbate the disease burden, particularly in patients with chronic obstructive pulmonary disease (45). The reason for this difference in results may be that, although unhealthy lifestyle habits increase the risk of illness, their impact may be relatively “diluted” in the face of the numerous challenges confronting the near-poor population. Furthermore, while medical insurance is a crucial tool for redistributing resources and alleviating the economic burden of disease among the poor (46), our study found that the type of medical insurance coverage did not significantly impact the risk of falling into medical impoverishment. This may be related to China's unique national conditions. In China, residents in rural areas primarily purchase urban and rural resident medical insurance, with coverage rates exceeding 90%. The subsidy standards for the same type of medical insurance are consistent, which may explain why its impact is not evident in this study. Additionally, descriptive analysis reveals that from 2016 to 2020, the number of uninsured individuals has grown, and concurrently, there has been an increase in the non-poor population. This trend suggests that there is potential for further enhancements in the medical insurance system (47).

Self-rated health status and the presence of chronic diseases significantly influence the probability of medical impoverishment among the near-poor population. Cross-sectional logit regression results indicate that individuals who perceive themselves as healthier have a significantly lower risk of falling into medical impoverishment. This reduction in risk is more pronounced among the near-poor than the non-poor, with the difference in risk reduction ranging between 7.3 and 20.9% points. The presence of chronic diseases also significantly impacts the risk of medical impoverishment among the rural populace (4, 40, 48). Specifically, the near-poor with chronic diseases face nearly an 11% point higher risk of medical impoverishment compared to their non-poor counterparts. This indicates that enhancing the health status of the near-poor population would result in significant benefits for poverty prevention.

Migrant status does not significantly influence the risk of medical impoverishment within the near-poor population. This conclusion is consistent across both cross-sectional and panel data analyses. This outcome diverges from research on immigrants in European countries (12), highlighting China's notable achievements in providing healthcare for migrants in recent years. Employment status, however, has a significant effect on the likelihood of the near-poor falling into medical impoverishment (4). Additionally, cross-sectional data from 2020 shows that the risk of medical impoverishment for the employed near-poor is 9% points lower than for their unemployed counterparts.

Marital status and household size may act as mediating variables affecting the risk of medical impoverishment among the near-poor population. Analysis of cross-sectional data indicates that household size has a substantial impact on the near-poor, with no significant effect on the non-poor. For the near-poor, large households with more than six members are less likely to experience medical impoverishment compared to smaller households with three or fewer members (40). Those who are married or cohabiting within the near-poor group are more susceptible to medical impoverishment than their single, divorced, or widowed counterparts. To investigate this seemingly contradictory phenomenon, we further examined the characteristics of the near-poor with household sizes of three or fewer who fell into medical impoverishment, finding that these individuals are predominantly older adult, aged between 50 and 70. Within this demographic, the proportions of older adult individuals aged 50–70 in households of three or fewer who suffered medical impoverishment vs. those in households of more than six members who did not were 69.37 and 39.16%, respectively, while the proportions of those not in employment were 36.04 and 19.25%, respectively. This contradiction may be attributed to household size and marital status possibly being intermediary variables, with the decisive factor for medical impoverishment still being the number of working-age individuals and their employment status.

In summary, China's near-poor population faces a high risk of falling into medical impoverishment. To further promote the development of the health program for poverty alleviation and consolidate the achievements of China's poverty alleviation efforts, the government should prioritize attention to the rural near-poor population. Specific improvements could include: (1) Perfecting a big data-based monitoring and early warning system to prevent relapse into medical impoverishment (49), increasing focus on key populations, and adjusting support measures in real-time based on monitoring data, thereby implementing targeted poverty alleviation and improving the efficiency of medical insurance fund utilization. This includes focusing on the rural near-poor, particularly older adult people with smaller family sizes, chronic disease patients, and those in poor health, by increasing the medical insurance fund's focus on key populations. This is directly related to the United Nations' SDGs of “No Poverty” and “Good Health and Wellbeing.” Identifying and supporting vulnerable groups through data-driven approaches is a crucial pathway to achieving the goal of “leaving no one behind.” (2) Building and perfecting a multi-level and multi-category medical security system, strengthening medical assistance for key populations, enhancing the risk pooling of outpatient services for medical insurance, increasing the compensation for outpatient care for chronic and special diseases (18), expanding the coverage of diseases and medical conditions, improving the basic medical insurance drug catalog, fully utilizing commercial and supplementary medical insurance, simplifying the medical insurance reimbursement process, and strengthening the promotion of medical policies to effectively enhance the near-poor population's resilience against health-related risks. This echoes the United Nations' SDGs of “achieving universal health coverage.” China's exploratory experiences, such as the dynamic adjustment of the medical insurance drug catalog and the reform of medical insurance payment methods, have important implications for other developing countries. (3) Implementing a holistic governance strategy, simultaneously improving health and employment assistance policies, and creating synergistic effects through multiple channels. The focus should be on enhancing the vocational skills of the rural near-poor, increasing their economic capacity through policy synergy, thus reducing the risk of medical impoverishment, and ensuring the sustainability of the health program for poverty alleviation. (4) Promote collaboration among the government, social organizations, communities, and individuals in providing social support to form a multi-level, multi-actor social support network. Encourage social organizations to participate in assistance for the impoverished population, mobilize the potential for mutual assistance within communities and individuals, and simultaneously leverage the government's leading and coordinating role. Engaging all parties to jointly participate in health-related poverty alleviation reflects the spirit of “Partnerships for the Goals” in the United Nations SDGs. This is an essential path to address complex social issues and represents a successful practice of China's “three-social linkage” mechanism.

This study has certain limitations. Although CFPS is a large-scale, complex, and nationally representative sample, it does not include areas with high concentrations of ethnic minorities, such as Xinjiang, Tibet, and Inner Mongolia, nor does it include Hong Kong, Taiwan, and Macau. Additionally, this study focuses on the rural population and does not analyze the situation of the urban population. This may result in the sample losing representativeness in certain regions. Moreover, as of now, the latest CFPS database is from 2020, which slightly lacks timeliness. Although a relative poverty perspective is adopted, the measurement dimension of poverty remains one-dimensional. Therefore, future research directions could include increasing studies on ethnic minority regions, conducting comparative research between urban and rural areas, and carrying out international comparisons. Simultaneously, research can be conducted from the perspective of multidimensional poverty.

## 5 Conclusions

This study highlights the critical importance of addressing the needs of the rural near-poor in health poverty alleviation initiatives. It confirms that in rural China, the near-poor experience a significantly higher risk of descending into medical impoverishment compared to the non-poor population. Additionally, the research reveals that the key factors contributing to the susceptibility of China's rural near-poor to medical impoverishment include employment status, self-rated health status, chronic disease presence, household size, and migrant status. These findings offer scientific support for improving China's healthcare insurance and other related systems, contributing to the progress of health poverty alleviation efforts. Future studies could expand upon this work by comparatively analyzing the factors affecting medical impoverishment among both urban and rural near-poor demographics, with a comprehensive consideration of the multidimensional aspects of social support networks and regional health resource distribution. This approach will be instrumental in formulating more precise health poverty alleviation strategies, advancing the implementation of precision medicine and social security measures, and ultimately reducing the overall risk of medical impoverishment.

## Data availability statement

Publicly available datasets were analyzed in this study. This data can be found at: http://www.isss.pku.edu.cn/cfps/index.htm.

## Author contributions

QF: Conceptualization, Data curation, Formal analysis, Methodology, Writing – original draft, Writing – review & editing. YC: Data curation, Formal analysis, Methodology, Validation, Writing – original draft. SY: Data curation, Formal analysis, Methodology, Validation, Writing – original draft. YW: Methodology, Writing – review & editing. WC: Conceptualization, Funding acquisition, Project administration, Writing – review & editing, Software. XX: Conceptualization, Funding acquisition, Project administration, Writing – review & editing.
