# Supplementary material for: Analysis of medical impoverishment and its influencing factors among China's rural near-poor, 2016–2020
Source: Front Public Health. 2024 May 16;12:1412536. doi: 10.3389/fpubh.2024.1412536 (PMC11137257; doi:10.3389/fpubh.2024.1412536)
Supplement: Supplementary file 1 [file Table_1.docx]

Supplementary Material

Supplementary Table 1 Variable Assignment and Baseline Characteristics of the Rural Chinese Population, 2016-2020

| Variables | Categories | Assignment | 2016 | | 2018 | | 2020 | |
| --- | --- | --- | --- | --- | --- | --- | --- | --- |
|  |  |  | Number(n) | Percent (%) | Number(n) | Percent (%) | Number(n) | Percent (%) |
| Household Economic Status | Poverty | 0 | 4,216 | 40.84 | 2,935 | 35.05 | 1,995 | 32.38 |
|  | Near-Poor | 1 | 2,005 | 19.42 | 1,763 | 21.06 | 1,263 | 20.50 |
|  | Non-Poor | 2 | 4,103 | 39.75 | 3,675 | 43.89 | 2,902 | 47.11 |
| Age (Years) | <35 | 1 | 2,836 | 27.47 | 1,798 | 21.48 | 1,569 | 25.47 |
|  | 35~65 | 2 | 5,661 | 54.83 | 4,826 | 57.64 | 3,293 | 53.45 |
|  | ≥65 | 3 | 1,827 | 17.70 | 1,748 | 20.88 | 1,299 | 21.09 |
| Gender | Female | 0 | 5,128 | 49.67 | 4,239 | 50.63 | 3,006 | 48.80 |
|  | Male | 1 | 5,197 | 50.33 | 4,134 | 49.37 | 3,154 | 51.20 |
| Marital Status | Single | 1 | 1,492 | 14.45 | 1,017 | 12.14 | 1,017 | 16.51 |
|  | Married/Cohabiting | 2 | 8,027 | 77.75 | 6,589 | 78.69 | 4,599 | 74.66 |
|  | Divorced/Widowed | 3 | 805 | 7.80 | 768 | 9.17 | 544 | 8.83 |
| Education Level | Elementary or Below | 0 | 6,012 | 58.24 | 4,598 | 54.92 | 2,939 | 47.71 |
|  | Secondary and Vocational School | 1 | 3,841 | 37.20 | 3,405 | 40.67 | 2,640 | 42.86 |
|  | College and Above | 2 | 472 | 4.57 | 369 | 4.41 | 581 | 9.43 |
| Employment Status | Employed | 1 | 7,679 | 74.38 | 6,254 | 74.70 | 4,619 | 74.98 |
|  | Other Status | 2 | 2,645 | 25.62 | 2,119 | 25.31 | 1,541 | 25.02 |
| Type of Medical Insurance Coverage | Without Medical Insurance | 0 | 681 | 6.60 | 590 | 7.05 | 573 | 9.30 |
|  | Basic Medical Insurance for Urban and Rural Residents | 1 | 9,125 | 88.38 | 7,269 | 86.82 | 5,036 | 81.76 |
|  | Other Medical Insurance | 2 | 519 | 5.02 | 514 | 6.13 | 551 | 8.95 |
| Smoking in the Past Month | No | 0 | 3,768 | 36.50 | 2,746 | 32.80 | 2,081 | 33.78 |
|  | Yes | 1 | 2,931 | 28.39 | 2,186 | 26.11 | 1,633 | 26.50 |
| Alcohol Consumption 3 Times a Week in the Past Month | No | 0 | 710 | 6.87 | 755 | 9.02 | 573 | 9.30 |
|  | Yes | 1 | 7,746 | 75.02 | 6,557 | 78.31 | 5,036 | 81.76 |
| Self-rated Health Status | Unhealthy | 1 | 1,869 | 18.10 | 1,061 | 12.67 | 551 | 8.95 |
|  | Average | 2 | 7,385 | 71.53 | 5,852 | 69.89 | 4,376 | 71.04 |
|  | Relatively Healthy | 3 | 2,939 | 28.47 | 2,521 | 30.11 | 1,784 | 28.96 |
|  | Very Healthy | 4 | 8,535 | 82.67 | 6,805 | 81.28 | 5,221 | 84.75 |
|  | Extremely Healthy | 5 | 1,789 | 17.33 | 1,567 | 18.72 | 939 | 15.25 |
| Presence of Chronic Diseases | No | 0 | 1,802 | 17.46 | 1,705 | 20.36 | 980 | 15.90 |
|  | Yes | 1 | 1,900 | 18.40 | 1,108 | 13.24 | 705 | 11.45 |
| Migrant Status | No | 0 | 3,260 | 31.57 | 3,066 | 36.62 | 2,411 | 39.13 |
|  | Yes | 1 | 1,954 | 18.92 | 1,337 | 15.97 | 1,057 | 17.17 |
| Household Size (Persons) | ≤3 | 1 | 1,409 | 13.65 | 1,157 | 13.82 | 1,008 | 16.36 |
|  | 4~5 | 2 | 8,659 | 83.87 | 7,011 | 83.73 | 5,224 | 84.80 |
|  | ≥6 | 3 | 1,665 | 16.13 | 1,362 | 16.27 | 937 | 15.21 |
| Total | | | 10,324 | 100.00 | 8,373 | 100.00 | 6,160 | 100.00 |
