# Supplementary material for: Analysis of medical impoverishment and its influencing factors among China's rural near-poor, 2016–2020
Source: Front Public Health. 2024 May 16;12:1412536. doi: 10.3389/fpubh.2024.1412536 (PMC11137257; doi:10.3389/fpubh.2024.1412536)
Supplement: Supplementary file 2 [file Table_2.docx]

Supplementary Material

Supplementary Table 2 Analysis of the Effects of Household Economic Status on Impoverishing Health Expenditures from 2016 to 2020 Using Conditional Fixed-Effects Multinomial Logit Models

| Variables | Model1 | Model 2 | Model 3 | Model 4 | Model 5 | Model 6 |
| --- | --- | --- | --- | --- | --- | --- |
| Household Economic Status (control group: Near-Poor) | | | | | | |
| Poverty | 28.900*** | 29.674*** | 28.207*** | 27.741*** | 30.805*** | 28.914*** |
|  | (0.213) | (0.202) | (0.078) | (0.084) | (0.219) | (0.219) |
| Non-Poor | -2.445*** | -2.418*** | -2.213*** | -2.255*** | -2.271*** | -2.436*** |
|  | (0.257) | (0.260) | (0.237) | (0.242) | (0.248) | (0.260) |
| Type of Medical Insurance Coverage (control group: Basic Medical Insurance for Urban and Rural Residents) | | | | | | |
| Without Medical Insurance | 0.331 | 0.348 |  |  |  |  |
|  | (0.447) | (0.442) |  |  |  |  |
| Other Medical Insurance | -0.560 | -0.558 |  |  |  |  |
|  | (0.453) | (0.465) |  |  |  |  |
| Household Size (control group: ≤3) | | | | | | |
| 4~5 | 0.884** | 0.904*** |  |  | 0.808** | 0.865** |
|  | (0.341) | (0.342) |  |  | (0.386) | (0.336) |
| ≥6 | 0.817** | 0.846** |  |  | 0.571 | 0.849** |
|  | (0.381) | (0.382) |  |  | (0.394) | (0.391) |
| Age (control group: ≥65) | | | | | | |
| <35 | 0.065 | <0.001 |  |  | 0.060 | <-0.001 |
|  | (0.701) | (0.606) |  |  | (0.628) | (0.705) |
| 35~65 | -0.726* | -0.783* |  |  | -0.733* | -0.770* |
|  | (0.430) | (0.409) |  |  | (0.389) | (0.442) |
| Gender (control group: Male) | | | | | | |
| Female | -2.797*** | -2.784*** |  |  | -1.918* | -2.804*** |
|  | (1.026) | (1.009) |  |  | (1.080) | (1.028) |
| Marital Status (control group: Married/Cohabiting) | | | | | | |
| Single | 3.317*** | 3.241*** |  |  | 3.518*** | 3.488*** |
|  | (1.084) | (1.076) |  |  | (1.072) | (1.053) |
| Divorced/Widowed | 0.834 | 0.859 |  |  | 1.079** | 0.833 |
|  | (0.619) | (0.625) |  |  | (0.512) | (0.611) |
| Education Level (control group: Elementary or Below) | | | | | | |
| Secondary and Vocational School | -0.783 | -0.747 |  |  | -0.828 | -0.802 |
|  | (0.655) | (0.626) |  |  | (0.685) | (0.651) |
| College and Above | -0.341 | -0.247 |  |  | -0.822 | -0.350 |
|  | (1.230) | (1.205) |  |  | (1.289) | (1.249) |
| Employment Status (control group: Employed) | | | | | | |
| Other Status | 0.455 | 0.451 |  |  | 0.623* | 0.447 |
|  | (0.305) | (0.310) |  |  | (0.332) | (0.304) |
| Smoking in the Past Month (control group: Yes) | | | | | | |
| No | 0.131 | 0.134 |  |  |  | 0.117 |
|  | (0.557) | (0.560) |  |  |  | (0.551) |
| Self-rated Health Status (control group: Unhealthy) | | | | | | |
| Average | -0.404 | -0.417 |  |  |  | -0.422 |
|  | (0.298) | (0.297) |  |  |  | (0.293) |
| Relatively Healthy | -0.967*** | -0.968*** |  |  |  | -0.966*** |
|  | (0.315) | (0.315) |  |  |  | (0.318) |
| Very Healthy | -1.580*** | -1.591*** |  |  |  | -1.590*** |
|  | (0.407) | (0.414) |  |  |  | (0.409) |
| Extremely Healthy | -1.525*** | -1.518*** |  |  |  | -1.540*** |
|  | (0.510) | (0.509) |  |  |  | (0.519) |
| Presence of Chronic Diseases (control group: Yes) | | | | | | |
| No | -1.090*** | -1.086*** |  |  |  | -1.096*** |
|  | (0.207) | (0.208) |  |  |  | (0.204) |
| Migrant Status (control group: Yes) | | | | | | |
| No | -0.430 | -0.386 |  |  | -0.265 | -0.428 |
|  | (0.315) | (0.310) |  |  | (0.316) | (0.318) |
| Year (control group: 2016) | | | | | | |
| 2018 | -0.066 |  |  | -0.197 | -0.044 | -0.070 |
|  | (0.155) |  |  | (0.225) | (0.142) | (0.153) |
| 2020 | 0.083 |  |  | -0.015 | 0.093 | 0.082 |
|  | (0.189) |  |  | (0.156) | (0.157) | (0.187) |
| N | 8099 | 8099 | 8099 | 8099 | 8099 | 8099 |
| F | 3723.55 | 4266.86 | 66654.92 | 34341.07 | 6641.79 | 3952.11 |
| P | <0.001 | | | | | |

*Note: Model 1: Two-way fixed effects (TWFE) with all variables (baseline model). Model 2: Individual fixed effects (IFE) with all variables. Model 3: IFE with only the core explanatory variable. Model 4: TWFE with only the core explanatory variable. Model 5: TWFE with the core explanatory variable and demographic variables. Model 6: TWFE with the core explanatory variable, demographic, and behavioral characteristic variables. The base outcome for this model is “No Experienced Medical Impoverishment”. The coefficients represent the change in the log-odds of experiencing medical impoverishment for a one-unit increase in the predictor variables, relative to the base outcome. ***p<0.001; **p<0.01; *p<0.05.*
