# Supplementary material for: Analysis of medical impoverishment and its influencing factors among China's rural near-poor, 2016–2020
Source: Front Public Health. 2024 May 16;12:1412536. doi: 10.3389/fpubh.2024.1412536 (PMC11137257; doi:10.3389/fpubh.2024.1412536)
Supplement: Supplementary file 3 [file Table_3.docx]

Supplementary Material

Supplementary Table 3 Logit Regression Analysis of the Influencing Factors of Medical Impoverishment among Rural Residents in China, 2020

| Variables | Near-Poor | | Non-Poor | |
| --- | --- | --- | --- | --- |
|  | Logit Regression | Marginal Effects | Logit Regression | Marginal Effects |
| Household Size (control group: ≤3) | | | | |
| 4~5 | -0.207 | -0.025 | 0.451 | 0.008 |
|  | (0.225) | （0.027） | (0.402) | （0.007） |
| ≥6 | -0.418* | -0.048 | -0.119 | -0.002 |
|  | (0.230) | （0.026） | (0.461) | （0.007） |
| Age (control group: ≥65) | | | | |
| <35 | -0.184 | -0.021 | -1.940** | -0.026* |
|  | (0.469) | （0.053） | (0.859) | （0.010） |
| 35~65 | -0.057 | -0.007 | -0.471 | -0.011 |
|  | (0.270) | （0.032） | (0.332) | （0.009） |
| Gender (control group: Male) | | | | |
| Female | 0.016 | 0.002 | 0.433 | 0.007 |
|  | (0.219) | （0.025） | (0.421) | （0.007） |
| Marital Status (control group: Married/Cohabiting) | | | | |
| Single | -0.722* | -0.071* | 0.487 | 0.011 |
|  | (0.410) | （0.034） | (0.807) | （0.021） |
| Divorced/Widowed | 0.461 | 0.061 | -0.809 | -0.011 |
|  | (0.282) | （0.040） | (0.762) | （0.008） |
| Education Level (control group: Elementary or Below) | | | | |
| Secondary and Vocational School | -0.139 | -0.016 | -0.143 | -0.002 |
|  | (0.221) | （0.025） | (0.290) | （0.005） |
| College and Above | -0.494 | -0.052 | -0.289 | -0.005 |
|  | (0.684) | （0.063） | (0.990) | （0.015） |
| Employment Status (control group: Employed) | | | | |
| Other Status | 0.709*** | 0.090*** | 0.460 | 0.009 |
|  | (0.201) | （0.027） | (0.402) | （0.008） |
| Smoking in the Past Month (control group: Yes) | | | | |
| No | 0.273 | 0.030 | 0.442 | 0.007 |
|  | (0.264) | （0.029） | (0.505) | （0.007） |
| Self-rated Health Status (control group: Unhealthy) | | | | |
| Average | -0.670*** | -0.117*** | -1.028** | -0.034* |
|  | (0.200) | （0.036） | (0.477) | （0.016） |
| Relatively Healthy | -1.225*** | -0.187*** | -1.950*** | -0.046** |
|  | (0.188) | （0.034） | (0.528) | （0.016） |
| Very Healthy | -1.582*** | -0.221*** | -2.052** | -0.047** |
|  | (0.343) | （0.043） | (0.864) | （0.018） |
| Extremely Healthy | -2.124*** | -0.258*** | -2.319*** | -0.049** |
|  | (0.416) | （0.038） | (0.793) | （0.015） |
| Presence of Chronic Diseases (control group: Yes) | | | | |
| No | -0.885*** | -0.120*** | -0.702** | -0.014* |
|  | (0.214) | （0.032） | (0.313) | （0.007） |
| Migrant Status (control group: Yes) | | | | |
| No | 0.256 | 0.028 | 1.755** | 0.017*** |
|  | (0.306) | （0.032） | (0.714) | （0.004） |
| _cons | -0.313 |  | -3.950*** |  |
|  | (0.390) |  | (0.874) |  |
| N | 1737 |  | 2937 |  |
| Pseudo R2 | 0.1682 |  | 0.2241 |  |
| P | <0.001 |  | <0.001 |  |
| F | 17.16 |  | 10.62 |  |

*Note: ***p<0.001; **p<0.01; *p<0.05. For ease of interpretation, each model reports both the logistic regression statistics and their marginal effects statistics, with standard errors in parentheses. The marginal effect coefficients are represented as dy/dx.*
